# The Diagnosis of Contracted or Granular Kidney

**Published:** 1888-05

**Authors:** A. D. Lake

**Affiliations:** Perrysburgh, N. Y.


### Original Communications.

### *THE DIAGNOSIS OF CONTRACTED OR GRANULAR KIDNEY.* *

By A. D. LAKE, M. D., Perrysburgh, N. Y.

*Mr. President and Gentlemen of the Alumni Association:*

The subject of my paper is the diagnosis of contracted or granular kidneys.

Among the more important diseases demanding our attention in practice, there are several, and this among the number, where our means of forming a certain early diagnosis are very limited. It seems to me this becomes a matter of serious import, when it is apparent that any large degree of success we may hope for in treatment, depends upon an early discovery of the conditions present.

* Read before the Alumni Association of the Medical Department of Niagara University, April 10, 1888.]

Only within a comparatively few years have the different forms of kidney affection, first described by Richard Bright, and receiving the name of that illustrious investigator, been distinguished in medical literature, or in practice, the one from the others. Even at this time, to some extent, the term Bright's disease, or chronic albuminuria, is made to cover, generally, chronic nephritis, contracted kidney, and lardaceous disease, when, as a matter of fact, not only the clinical history, but the pathological conditions, are so far dissimilar as to fully warrant an entire change in the nosology.

One important point in the differentiation of these diseases consists in the ease with which we recognize the existence of two of them, and the great difficulty, from the obscurity of symptoms, in determining the existence of the other, until a late stage in its progress is reached.

The symptomatology of chronic nephritis, and of amyloid degeneration, is so well pronounced and characteristic, that a diagnosis is readily made at any period of the disease; but, on the other hand, contracting kidney is an affection so insidious in its approach, that it may have reached a serious degree of development before the patient becomes aware of any kind of symptom, that his health is in any way impaired. No doubt, patients die suddenly of this disease, or after a short illness, preceded only, but perhaps for a long time, by slight derangements of the digestive organs, apparently so trivial, that they have not sought medical advice.

I believe that many of those obscure cases, resulting fatally, in which we are unable to arrive at a positive diagnosis, are contracted kidney, and I also believe that many cases of fatal apoplexy are preceded, perhaps for years, but unrecognized, by this disease.

Ziemssen says: "In the larger proportion of cases, striking symptoms, indicative of the disease, precede its fatal termination, and are occasionally prolonged over a period of several years. It is, undoubtedly, in most of cases, a disease of long duration, and it must have existed for some time before a certain diagnosis can be made. It is very likely the first beginning is never discovered."

Permit me to call your attention, briefly, to the more common symptoms, taken in the order of their observance :

1. Increased quantity of urine, which in no way seems to depend upon the quantity of fluid drank, and is at all times persistent throughout the disease, existing sometimes to such an extent as to lead the patient to think he has diabetes. This condition is an important factor in the diagnosis of contracting kidney.

2. Attacks of palpitation, vertigo, and difficulty of breathing, which are probably due to enlargement of the heart. One is quite likely, on discovering this condition of the heart, to bring his investigations to a close, and be satisfied with a diagnosis of valvular disease, until the appearance of more characteristic signs cause him to change his opinion.

3. Headache. This is an early symptom, and very persistent, not usually severe, and the patient seems to get accustomed to it, so far that he may not complain of it, but, when asked, says he has headache, and has suffered from it for a long time.

4. Sleeplessness. In the cases coming under my observation, this was a very prominent symptom: The patients often going to sleep readily on retiring, but awakening to pass urine, finding themselves unable to go to sleep again.

5. Disordered vision, probably not occurring until the disease is well advanced.

6. After the affection has reached a certain stage, but before this it may have existed for years, disorders of digestion make their appearance. The appetite fails, and the patient has severe pain after meals, and acquires a strong dislike for a meat diet. In no other disease is there such entire deprivation of relish for food, patients frequently being nauseated at even hearing the preparations for meals.

At this period the most troublesome thirst exists.

Following the disordered digestion, ensue emaciation, anæmia, and loss of strength, the skin assumes a dirty, faded color. In most of cases, the disease reaches this point before the patient becomes aware that he has any serious sickness. His spirits now become low; he is gloomy and moody.

Most patients do not exhibit any dropsy throughout the disease, but in many cases, by careful examination, slight œdema may be discovered along the spine of the tibia, or about the ankle. Diarrhœa and vomiting are apt to occur in the later stages. This condition is quite likely to be followed by uræmic coma, terminating in death.

An examination of the urine, in contracting kidney, is very apt to be misleading. Throughout the whole course of the disease, urine is occasionally, and for a short time, excreted, which, in all respects, is like that secreted by healthy kidneys. The urine is always increased in quantity, and this is more marked during the night than during the day. The color is pale yellow. The specific gravity is below normal. The reaction is slightly acid. Albumin is irregularly present in small quantity, frequently requiring delicate tests and most careful manipulations to discover its presence. At times it entirely disappears—this is very likely to be the case if patients are in bed any length of time—to again make its appearance when they are up and moving about. The urine deposits only a very small amount of sediment.

But little aid in diagnosis is to be expected from the use of the microscope, except it be in the very last stage of the disease. Possibly, before this time, a few scattered hyaline casts may be discovered.

I have gone rapidly over the more pronounced and prominent symptoms of contracted kidney, for the purpose of comparison of the same with the condition present in the following case in practice:

Mrs. C., age sixty-eight. I had seen her occasionally for two years before the conditions herein narrated existed, being called upon to prescribe for vertigo and congestive headaches. She had, twice I think, fallen while at work, remaining for a few minutes unconscious. I was inclined to regard these attacks as threatened apoplexy, and prescribed accordingly. I am certain now that the predisposing cause of the attacks was either commencing, or existing, granular degeneration of the kidneys. In November, 1885, she began to lose strength and appetite. I had not given her case attention for several months, but was called to see her at this time. She was pale and anæmic, troubled with difficult breathing, upon making any extraordinary exertion. Her skin presented a pale and sallow appearance. Her headache had been for some time unusually severe, and she complained of her inability to read, because, as she said, the "words all ran together," and spots appeared before her eyes. Her appetite was so very poor, that it was with the utmost difficulty she could be induced to eat anything. This was especially marked as regarded milk, or animal food of any kind. From an examination made at this time, I became satisfied she had enlargement of the heart. By inquiry, I ascertained she was passing a large amount of urine, and that this had been the case for some time. By measurement, the quantity was found to be seven pints in twenty-four hours. The urine was pale, and of lessened specific gravity. An examination for albumin, by heat and nitric acid, showed the very slighest cloudy appearance, of which I was uncertain, until comparison with another specimen, which had not been submitted to test.

A daily examination of the urine was made, and continued for a period of three weeks. At times, there was a slight increase in the quantity of albumin, while at others it entirely disappeared. At this time, another physician saw her in consultation, and was unable to satisfy himself, from our test of the urine, that it contained albumin, nor could I, except from my repeated examinations.

I now sent a sample of her urine to Dr. Walker, an accomplished microscopist of Franklinville, Cattaraugus county, who wrote me as follows:

DEAR DOCTOR—Yours of the 28th inst., also sample of urine, received. I have made repeated examinations of the sediment, and can find no casts of the uriniferous tubules present. I find the reaction acid, specific gravity 1,018, and no deposit of *albumin* with heat or nitric acid.

Should the symptoms which lead you to suspect kidney disease continue, another examination of the urine, after a few weeks, might reveal casts. Very truly yours,

H. D. WALKER.

I had become convinced, by the steady appearance of albumin, although in such small quantity, and the constantly increasing anæmia, with other symptoms before stated, that my patient had granular kidney. At this time, she was so weak as to be compelled to keep the bed, only sitting up occasionally.

After she went to bed, the albumin almost entirely disappeared. It was at this time I sent the sample of urine to Dr. Walker. After a while she began to slowly gain strength, her appetite returned, and she was able to sit up, and soon to move about the house. Slight œdema of the ankles was now noticed, and I again discovered albumin in the urine. Her improvement continued, and after some weeks she was well enough to resume her usual occupations, the marked dirty pallor of the skin continuing, and albumin persisting in the urine. She now passed from my care and observation for several months. In about a year from the first appearance of the symptoms which led me to suspect the existence of granular kidney, I was again called to see her. She had, in the interval, been in fairly comfortable condition, but, whilst on a visit to Dunkirk, had become suddenly worse, and at this time there was an alarming increase of debility, with an entire loss of appetite, an occasional attack of vomiting, headache, and great disturbance of vision. She was very pale, and would stagger on walking across the room. She was still passing a large quantity of urine, which, on examination, was found to contain albumin in larger quantity than at any time before. There was considerable œdema of the ankles and some puffiness of the lower eyelids.

At this time I sent another sample of her urine to Dr. Walker, and he wrote me as to the result of his examination as follows:

Dear Doctor—I have made an examination of the urine you sent me, with the following result: Reaction acid, specific gravity 1,010; by nitric acid and heat, a well-marked deposit of albumin; by the microscope, a copious deposit of uric acid, and also plenty of well-marked granular casts were found, several of the latter being found in each drop of the sediment examined.

There can be no doubt about the case, from this examination. Would like to know her further history. Very truly yours,

H. D. WALKER.

The progress of her case after this was rapidly downward, and she died in about six weeks in uremic coma. During the whole course of her long sickness, her urine was above the normal amount in quantity, and at no time was there extensive dropsy.

I am convinced that it would have been impossible, until within a few weeks of her death, to have made a diagnosis of contracted kidney by either an examination of the urine for albumin, or by the microscope for casts.

Nevertheless, in establishing the fact as to the existence of this disease, it seems to me that repeated and careful tests of the urine for albumin stand first in importance. I have found, by the usual tests of heat and nitric acid, the urine of a patient may not appear different than normal urine, but by a comparison of that to which the tests have been applied, with the same urine without the tests, a slight opacity may be discovered in the former. A repetition of the examination next day may show a disappearance of the opacity, or an increase of the same. Following it up for a week, or, if necessary, for a month, never failing to make daily examinations, it becomes a very important factor in arriving at a diagnosis.

I have been so far impressed with the truth of this, that I make far more frequent examinations of my patients' urine than formerly, and find it important to make those examinations more carefully and more critically, but never expecting, in true granular kidney, that albumin will be found in any gross quantity.

The increased quantity of urine, persisting for some time, should always excite one's suspicions. The headache and marked dirty pallor are signs of importance; also the loss of appetite, and steadily increasing anæmia and muscular weakness.

There is undoubtedly a strong tendency to hemorrhage, and especially to cerebral hemorrhage. It is to be believed that this predisposition is shown in many cases by certain symptoms, such as vertigo, attacks of momentary unconsciousness, etc., which are surely to be taken into consideration in the diagnosis. A large percentage of all cases of granular kidney are accompanied, at some stage of the affection, by enlargement of the heart. The fact that this condition exists without valvular lesion, is a valuable diagnostic sign.

I believe little assistance is to be expected from the use of the microscope. It seems to me we are likely to be led astray by placing much reliance upon it.

In other forms of Bright's disease, its aid is invaluable; but here the diagnosis must rest upon a careful study and analysis of all the symptoms, with regular and frequent examinations of the urine for albumin, and, above all, a careful examination and study of the persistent condition of what may be known as indefinite illness, occurring in an individual over fifty years of age, where the patient is pale, the pallor of a dirty and slightly greenish hue, and he complains of some pain and inconvenience after eating; often has slight headache and irregular muscular pains; is easily tired out; has sleepless nights; his respiration somewhat hurried on exertion; is subject to dizzy attacks; is compelled to get up at night more often than formerly to void his urine, but, withal, still able to continue at his business, and is not easily convinced he is seriously sick. I believe this to be a fair picture of the early stage of many cases of the disease under consideration.

I am led to believe that contracted kidney is a comparatively common disease, and that it demands, at our hands, more careful study, looking to some means, not now at our command, whereby we may more certainly discover its existence at an early stage in its progress. If this paper shall, in the least, contribute to this end; if it shall invite attention and thought, and, perhaps, investigations on the part of others far better fitted for the work than the writer, then it will have abundantly fulfilled its mission.

